# Knowledge and attitude among Egyptian medical students regarding the role of human papillomavirus vaccine in prevention of oropharyngeal cancer: a questionnaire-based observational study

**DOI:** 10.1038/s41598-025-86853-8

**Published:** 2025-01-30

**Authors:** Mohammed. N. Abdelaziz, Abdullah Hefnawy, Hajer Azzam, Omar Reisha, Omar Hamdy

**Affiliations:** 1 Medical Intern, Faculty of Medicine, Mansoura University, Al Mansurah, Egypt; 2 Medical student, Integrated medical program, Faculty of Medicine, Mansoura University, Al Mansurah, Egypt; 3 Lecturer of Surgical Oncology, Oncology Centre, Mansoura University, Al Mansurah, Egypt; 4 Medical Intern, Faculty of Medicine, Mansoura University, Egypt, Arab Republic of Egypt- Al-Daqahlia Governorate, Al Mansurah, 35516 Egypt

**Keywords:** HPV, Vaccination, Oropharyngeal cancer, Awareness, Cancer prevention, Cancer, Health care, Medical research

## Abstract

Squamous cell carcinomas in several anatomical sites are caused by human papillomaviruses (HPV), and oncogenic double-stranded DNA viruses. There are about 200 genotypes; HPV16 is the most often occurring variant. Potential ways of infection are skin warts, sexual activity, exposure, immunization, or oral sex. The growing incidence of OPSCC in Western countries makes vaccination increasingly vital. The FDA has approved the 9-valent vaccination as an OPSCC prophylactic agent. Still, political will, inadequate financing, and inadequate infrastructure help to explain the slow dissemination of anti-HPV vaccination. This study sought to evaluate Egyptian medical students’ knowledge, awareness, and attitude toward the advantages of HPV vaccination to prevent HPV-associated OPC. The study was a cross-sectional questionnaire-based study consisting of 696 participants from the first to fifth-year students enrolled in any Egyptian medical school registered in the academic year 2023–2024, specifically from June to August 2024, except non-medical, graduate, and non-Egyptian students who met the exclusion criteria. We privately gathered answers via colleagues and electronically via online Google forms posted on social media groups. This study concentrated on the poor knowledge of HPV among Egyptian medical students, particularly urban male students with a mean age of 21.72 ± 1.6 enrolled in clinical years mostly in Cairo, Sharqia, and Gharbia governorates. There was a clear fair attitude regarding the HPV vaccination, especially among urban male students with a mean age of 21.64 ± 1.65 enrolled in clinical years mostly in Cairo, Sharqia, and Alex governorates. Notably, only 7.7% of the students enrolled in the study knew the link between HPV and OPC. However, only 28.5% of participants have received the vaccine. The students said that lack of awareness (82.4%) was the most important obstacle to vaccination; followed by cultural attitudes (44.5%), vaccine accessibility (42.7%), and vaccine cost (41.3%). Ultimately, it was found that Egyptian medical students—especially those enrolled in clinical years in the governorates of Cairo, Sharqia, and Gharbia—have a low degree of knowledge of HPV-related oropharyngeal cancer and its vaccination role. Although preclinical students’ knowledge had greatly improved, the limited awareness—especially among clinical students—was concerning.

## Introduction

Human papillomaviruses (HPV) are double-stranded DNA, simple, carcinogenic viruses devoid of an envelope that can cause squamous cell carcinomas in the respiratory, genitourinary, gastrointestinal, and cutaneous areas (papilloma and warts)^1,2^. There are almost two hundred different genotypes of HPV known. Mucosal HPV strains are categorized as either high or low-risk variants according to their capacity to cause cancer. Twelve strains have been considered as carcinogens. About 80% of cases of oropharyngeal squamous cell carcinoma in the high-risk HPV category are HPV16, the most often occurring variant. With about 3% of all affected cases, HPV-18 is the second most often occurring strain. Lesser proportion of patients had other genotypes including HPV33, HPV35, and HPV58^3–5^. HPV infection can strike one from skin warts, sexual activity (which can produce genital warts), or being exposed during normal vaginal delivery, immunization, or oral sex. Rarely occurring in newborns with oral HPV infection, young recurrent respiratory papillomatosis may be a cause of concern. While food and medical supplies can spread HPV, contaminated objects rarely have an impact on the external genitalia^6^.

The incidence of oropharyngeal squamous cell carcinoma (OPSCC) has dramatically risen in Western nations during the past few years, which leads to attribution to HPV infection. Among the younger population especially, this rise is remarkable^7^. These elements have made vaccination more and more important since they can effectively stop a significant portion of the risk for cancers. OPSCC prophylaxis has lately been approved by the Food and Drug Administration (FDA) as a use for the 9-valent vaccinations in both human and animal species. But expected to finish by 2024, the results of the current phase 3 trial will decide how effective the 9-valent vaccination is for men^8^. The latest HPV virus-like particles (VLPs) vaccinations, designed to target HPV 16/18, can prevent over 90% of oropharyngeal cancers positive for HPV. The World Health Organization (WHO) advises as follows^9^. Implementing a simplified screening program presents great difficulties, which emphasizes the need to advance primary prevention through effective risk factor control and immunization campaigns. Immunization has the potential to help to mostly prevent HNSCC, even if its exact degree of influence is yet unknown.

The difficulty in spreading HPV vaccines in this region stems from financial restraints, poor infrastructure for immunizing teenagers, competition with other top-priority vaccines, and a dearth of accurate information on the frequency of HPV-related diseases. Still, the main obstacle is insufficient authorities’ will, usually ascribed to cultural and religious sensitivity^10^. The above-mentioned problems could reduce the effectiveness of HPV vaccination campaigns in the area^11^. Particularly among medical students, thorough academic studies have repeatedly shown that healthcare professionals, including those in oral cancer-related fields, lack knowledge about HPV^12^. Testing how well undergraduate medical students can detect early signs of oral cancer and precancerous lesions and quickly refer patients with these issues is thus becoming more and more crucial.

As more nations implemented national HPV vaccination programs, vaccine acceptance became a focus of research. One of the well-reported indicators of HPV vaccine acceptability among the various parameters under study is awareness of HPV and the HPV vaccine^13^. Additionally, doctor recommendations are a significant factor in HPV vaccine uptake, with reports indicating that uptake can increase by up to 80%^14,15^.. Understanding disease transmission and prevention knowledge, community social support, family health history, including genetics, motivation, and health literacy, socioeconomic status, educational attainment, financial circumstances, and cultural healthcare resources are the primary factors that are crucial in individual health efforts to prevent and respond to illness^16^. HPV and HNC can be prevented through education, preventative measures, and HPV vaccination. Because of concerns regarding the safety of this vaccine, most parents are hesitant to have their siblings vaccinated. The incidence of head and neck cancer (HNC) will therefore be reduced with strong evidence of sound understanding of the relationship between HPV and vaccination in the development of this tumor^17^. Additionally, knowledge, among many other variables, is critical in making the HPV vaccine accessible to the general public, despite its importance in cancer prevention. More demand for and accessibility to cancer-prevention vaccines would be justified with a more informed population. So, this study aims to evaluate Egyptian medical students’ opinions on the efficacy of HPV vaccination in preventing OPSCC as well as their degree of knowledge on HPV and its vaccination.

## Methods

### Population and study design

We used a questionnaire data-collecting approach for our study in Egypt. Specifically, from June to August 2024, the study cohort comprised Egyptian students registered in any medical institution during the academic year 2023–2024. Except for non-medical, graduate, and non-Egyptian students who fit the exclusion criteria, the study comprised students from all five academic years. Students registered in the first and second academic years were referred to as “preclinical students.” Conversely, we classified third-, fourth-, and fifth-year students as “clinical students.” The sample size was calculated through an online tool accessible at (https://www.openepi.com/SampleSize/SSPropor.htm). Malik Sallam et al.^12^ reported a probability of high HPV knowledge, at 21℗. Under a 95% confidence interval and a 5% margin of error, the minimum sample size needed is 254. A 40% projected drop rate produces a final minimum sample size of 356. Students were gathered using a convenience sampling technique; each medical school sent representatives. Every group member sent the questionnaire using an instant messaging tool like WhatsApp, Facebook, or Messenger Inc., together with a direct hyperlink created by the survey management tool Forms developed by Google Inc. The participants carefully answered every question before sending the online form to the web server. All study procedures were performed by the Declaration of Helsinki.

### Data collection

A review of current literature^12,18–20^ led a panel of public health and oncology experts to establish the validity of the three-section structured questionnaire consisting of demographic information, knowledge about HPV and oropharyngeal cancer, knowledge, and attitudes toward HPV vaccination. To confirm the simplicity and clarity of the questionnaire’s completion, we ran a pilot study including thirty-five Egyptian medical students. We then deleted from the final analysis the results of this pilot study. The Cronbach alpha test helped us to evaluate internal consistency and degree of dependability. Three attitude scales and seventeen knowledge questions display an acceptable value (a = 0.75). The questionnaire consisted of three sequential questions to investigate the attitudes and knowledge of medical students about the HPV vaccination and HPV-related OPSCC. *The first section* evaluated the attributes and covariates of the participants, such as age, gender, marital status, geographical location (rural or urban), nationality, study area, and academic level (first to fifth years). After the first section, we asked participants, “Have you heard about HPV?” to assess their level of knowledge of the virus. Participants who responded positively were sent to *the second session*, which comprised seven fundamental HPV-OPSCC knowledge questions (yes/no) and multiple-choice questions (MCQs). After selecting “no,” we finished the questionnaire. “Have you heard about the HPV vaccine?” was included among the screening questions meant to gauge knowledge and attitudes about vaccination. After confirming their familiarity with the HPV vaccination, participants progressed to *the third section* (six questions about their impressions of their knowledge of the vaccination). These questions consisted of two MCQs, one yes/no question, and three statements on the degree of their practice 5-point Likert scale (1 = strongly disagree; 5 = strongly agree). The last multiple-choice question on the survey asked the participants about their sources of information on the HPV virus vaccination.

### Statistical analysis

IBM SPSS Statistics for Windows, Version 25.0 Armonk, NY: IBM Inc was applied to data analysis. Frequencies and percentages helped to describe categorical data. Usually following normality graphs, continuous data were described using mean and standard deviation. Knowledge and attitude scores were computed and arranged into good or poor for knowledge and suitable or inappropriate for attitude. The cutoff point was decided upon as the median (75%). Continuous data was compared using the independent t-test. Frequency was compared using the chi-square test. Continuous data correlations were tested using Pearson. Interesting predictors of good knowledge were investigated using binary logistic regression.

## Results

**Fig. 1:**
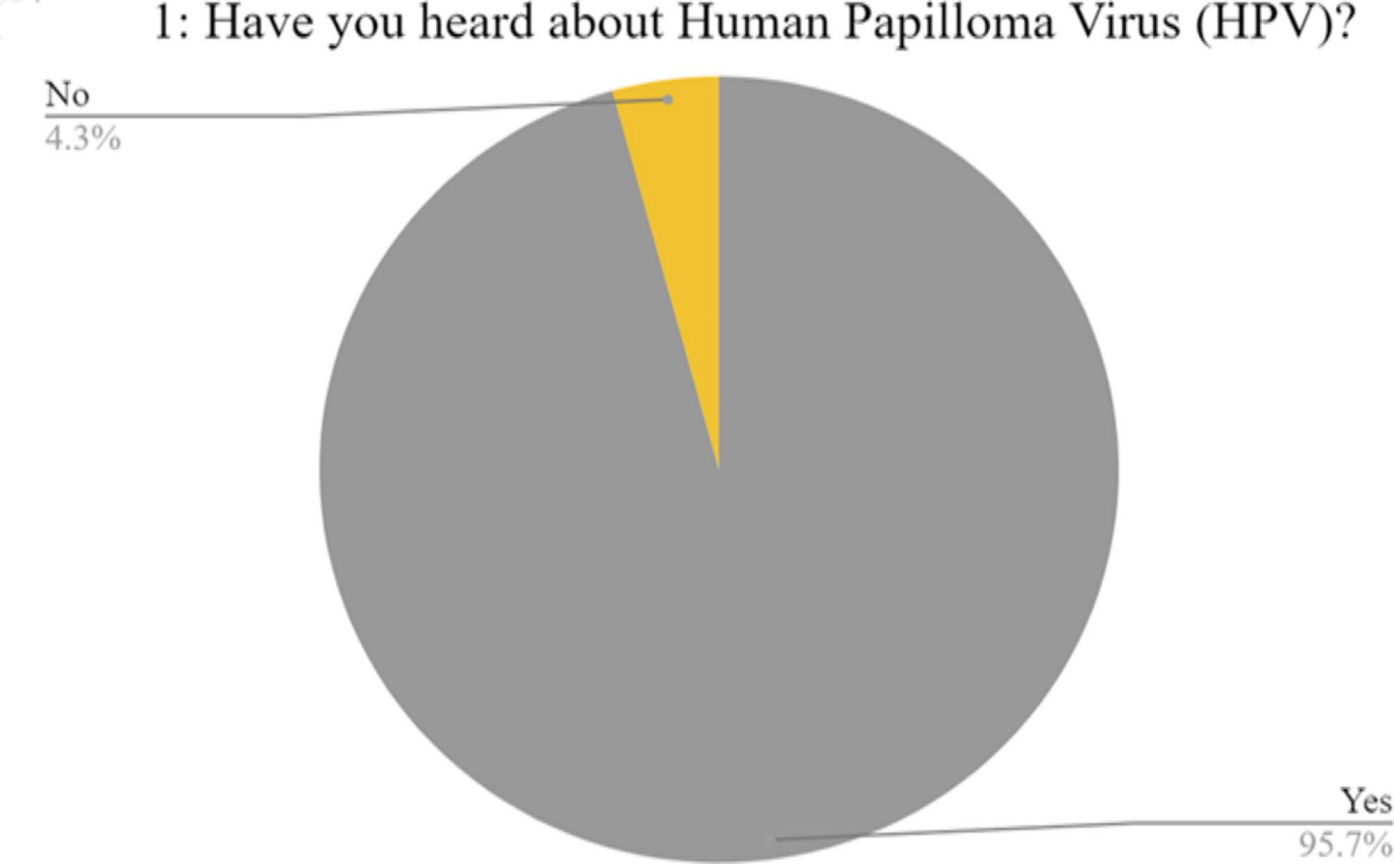
Pie chart illustrating the proportion of the population ignorant of HPV.

Our sample consisted of 696 participants from the first to fifth year with the majority (75.4%) enrolled in clinical years (Table 1). Their age ranged from 17 to 25 with a mean of 21.7 (1.6). 412 (59.2%) were males and 284 (40.8%) were females. The Governorates of Cairo, Sharqia, Gharbia, Dakahlia, and Alexandria represented the majority with frequencies of 193 (27.7%), 172 (24.7%), 92 (13.2%), 84 (12.1%) and 82 (11.8%), respectively. 524 (75.3%) were from urban residences and 172 (24.7%) were from rural residences. Most participants, 641 (92.1%), were single. 666 (95.7%) of participants said that they previously heard of HPV and 30 (4.3%) students who hadn’t heard about the virus were directed to the end of the questionnaire (Fig. 1).

**Table 1:** Demographics of the participants and their distribution among levels of HPV knowledge and attitude toward HPV vaccine.

Students characteristics	Studied group (696)	Poor knowledge (*n* = 652)	Good knowledge (*n* = 14)	*p*-value	Inappropriate attitude (*n* = 195)	Appropriate attitude (*n* = 418)	*p*-value
Age ➢ Mean ± SD	21.7 ± 1.6	21.72 ± 1.6	20.64 ± 1.6	0.01*	21.86 ± 1.58	21.64 ± 1.65	0.13
Gender ➢ Male ➢ Female	412 (59.2%) 284 (40.8%)	387 (59.4%) 265 (40.6%)	7 (50%) 7 (50%)	0.48	114 (58.5%) 81 (41.5%)	268 (64.1%) 150 (35.9%)	0.18
Year of Study ➢ Pre-clinical ➢ Clinical	171 (24.6%) 525 (75.4%)	147 (22.5%) 505 (77.5%)	8 (57.1%) 6 (42.9%)	0.002*	47 (24.1%) 148 (75.9%)	98 (23.4%) 320 (76.6%)	0.86
Region of study ➢ Cairo ➢ Alex ➢ Assiut ➢ Aswan ➢ Beheira ➢ Benha ➢ Beni Suef ➢ Damietta ➢ Suez ➢ Gharbia ➢ Ismailia ➢ Luxury ➢ Dakahlia ➢ Menofia ➢ Minia ➢ Port Said ➢ South Sini ➢ Sharqia	193 (27.7%) 82 (11.8%) 14 (2%) 1 (0.1%) 4 (0.6%) 1 (0.1%) 2 (0.3%) 12 (1.7%) 1 (0.1%) 92 (13.2%) 3 (0.4%) 1 (0.1%) 84 (12.1%) 24 (3.4%) 2 (0.3%) 7 (1%) 1 (0.1%) 172 (24.7%)	175 (26.8%) 76 (11.7%) 14 (2.1%) 1 (0.2%) 4 (0.6%) 1 (0.2%) 2 (0.3%) 11 (1.7%) 1 (0.2%) 87 (13.3%) 3 (0.5%) 1 (0.2%) 80 (12.3%) 24 (3.7%) 2 (0.3%) 7 (1.1%) 1 (0.2%) 162 (24.8%)	9 (64.30%) - - - - - - 1 (7.1℅) - 1 (7.1%) - - 1 (7.10%) - - - - 2 (14.3%)	0.7	35 (17.9%) 23 (11.8%) 3 (1.5%) 1 (0.5%) 2 (1%) - 2 (1%) 5 (2.6%) 1 (0.5%) 32 (16.4%) 2 (1%) 1 (0.5%) 28 (14.4%) 10 (5.1%) 1 (0.5%) 4 (2.1%) - 45 (23.1%)	143 (34.2%) 51 (12.2%) 11 (2.6%) - 2 (0.5%) - - 5 (1.2%) - 36 (8.6%) 1 (0.2%) - 48 (11.5%) 10 (2.4%) 1 (0.2%) 3 (0.7%) 1 (0.2%) 106 (25.4%)	< 0.001*
Residence ➢ Urban ➢ Rural	524 (75.3%) 172 (24.7%)	490 (75.2%) 162 (24.8%)	10 (71.4%) 4 (28.6%)	0.75	141 (72.3%) 54 (27.7%)	326 (78%) 92 (22%)	0.12
Marital status ➢ Single ➢ Engaged ➢ Married ➢ Divorced	641 (92.1%) 51 (7.3%) 3 (0.4%) 1 (0.1%)	602 (92.3%) 47 (7.2%) 2 (0.3%) 1 (0.2%)	11 (78.6%) 3 (21.4%) - -	0.257	182 (93.3%) 12 (6.2%) 1 (0.5%) -	382 (91.4%) 34 (8.1%) 1 (0.2%) 1 (0.2%)	0.68

**Fig. 2:**
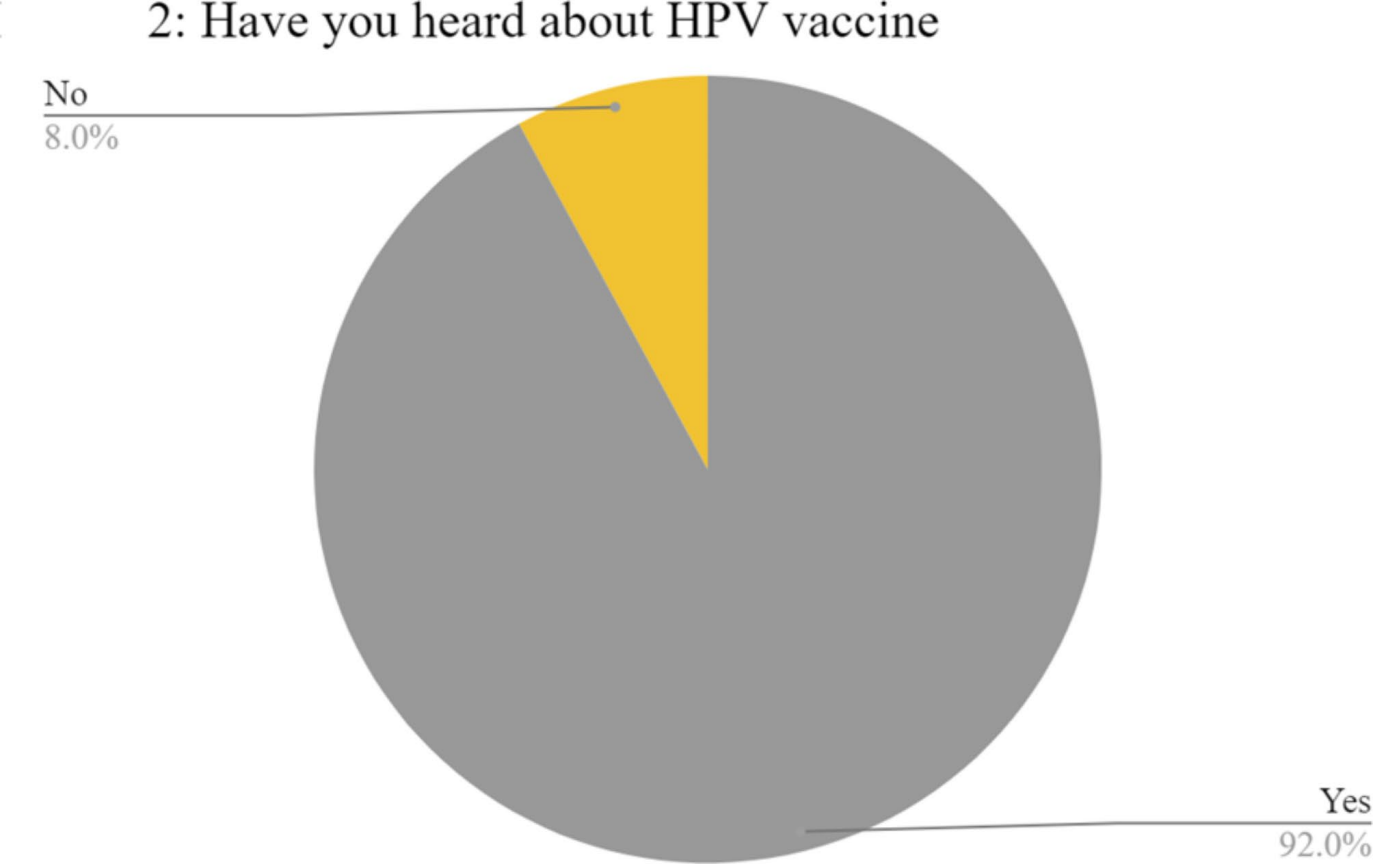
Pie chart displaying the proportion of people not knowing about the HPV vaccination.

Most of them, 652 (97.9%), had poor knowledge about HPV (Table 1). Their mean age was 21.72 (1.6). Most of them were males (59.4%), enrolled in clinical years (77.5%), from Governorates of Cairo (26.8%), Sharqia (24.8%), Gharbia (13.3%), urban residents (75.2%), and single (92.3%). Only age, year of study, and region of study significantly affected the level of knowledge, *p* = 0.01, 0.002, 0.03, respectively. The major defect in knowledge about HPV was found in the number of HPV types. 76.4% of medical students had limited awareness about HPV types which is considered the main knowledge defect. According to the correct mode of transmission, 85.7% chose sexual transmission, skin-to-skin (57.8%), blood transmission (31.8), and airborne (9.5%). Regarding HPV-related diseases, cervical cancer, and genital warts come at the top of student correct answers, (78.4%) and (13.7%) respectively followed by skin warts, OPC, and penile cancer, (9.9%),(7.7%), and (7.2%) respectively (Table 2). According to binary logistic regression, preclinical students had better HPV knowledge than clinical students (95%CI, *p* = 0.005). Of 666 who have heard about HPV, 613 (92%) have heard about the HPV vaccine (Fig. 2).

**Table 2:** Summary of HPV and HPV vaccine knowledge answers.

Knowledge question	Incorrect answer	Correct answer
How many types of HPV exist? > 200 types	509 (76.4%)	157 (23.6%)
How is HPV transmitted?		
➢ Sexual	95 (14.3%)	571 (85.7%)
➢ Blood	454 (68.2%)	212 (31.8%)
➢ Skin to skin	281 (42.2%)	385 (57.8%)
➢ Airborne	603 (90.5%)	63 (9.5%)
Can HPV infection lead to cancer?	52 (7.8%)	614 (92.2%)
Can HPV infection be asymptomatic?	127 (19.1%)	539 (80.9%)
Which of the following diseases can HPV cause?		
➢ Cervical cancer	144 (21.6%)	522 (78.4%)
➢ Genital warts	575 (86.3%)	91 (13.7%)
➢ Skin warts	600 (90.1%)	66 (9.9%)
➢ Oropharyngeal cancer	615 (92.3%)	51 (7.7%)
➢ Penile cancer	618 (92.8%)	48 (7.2%)
Which HPV types are most commonly associated with cancer?	269 (42.8%)	360 (57.2%)
What age groups should receive the HPV vaccine?		
➢ Pre-teens (9–12 years)	389 (63.5%)	224 (36.5%)
➢ Teenagers (13–17 years)	484 (79%)	129 (21%)
➢ Young adults (18–26 years)	444 (72.4%)	169 (27.6%)
➢ Adults (27–45 years)	513 (83.7%)	100 (16.3%)

**Fig. 3:**
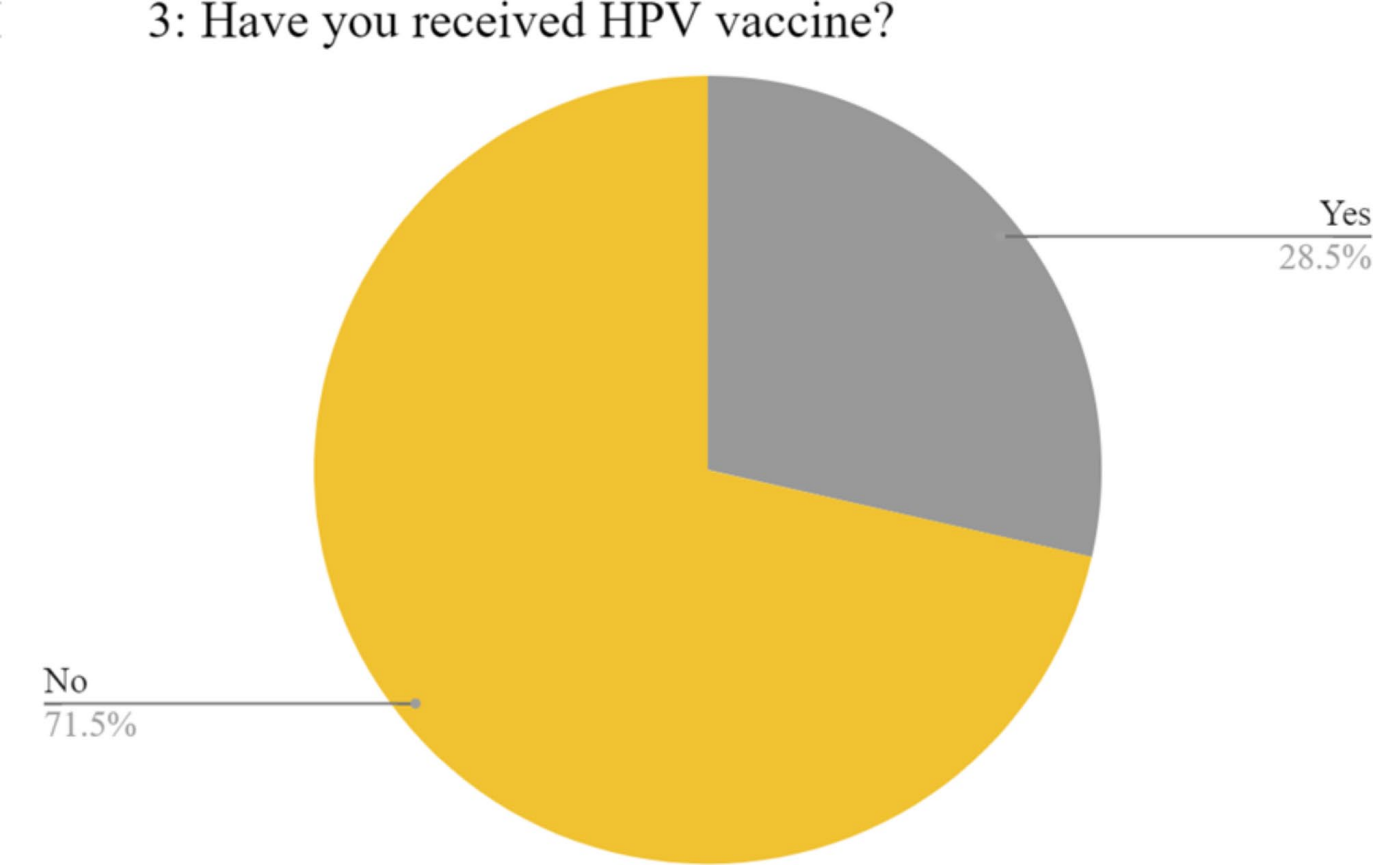
Pie chart illustrating vaccination acceptance rate.

**Fig. 4:**
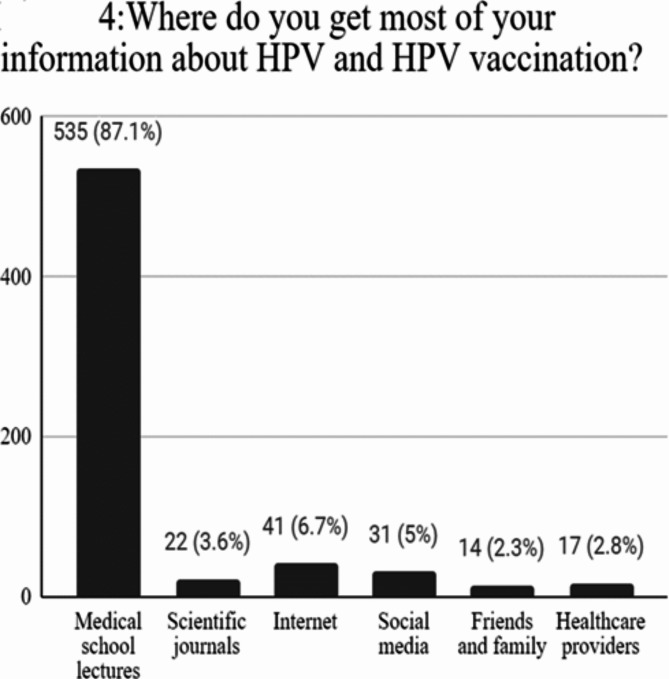
The bar chart displays the sources of HPV knowledge including information on its vaccination.

**Fig. 5:**
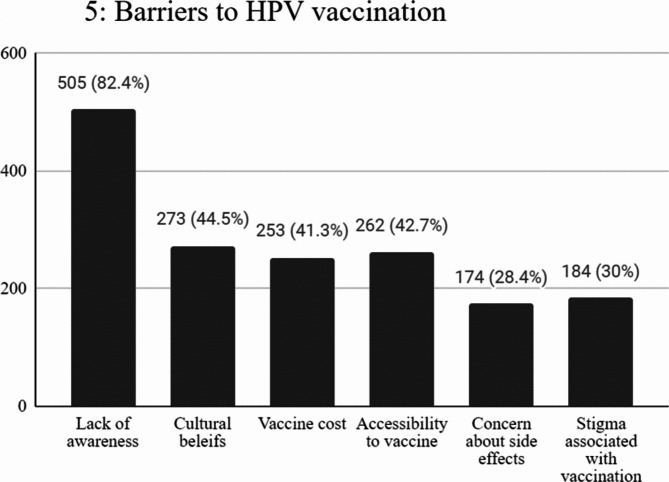
The bar graph reveals the obstacles to the HPV vaccination.

Most of them, 418 (68.2%), reported appropriate attitudes towards vaccination with a significant effect of the region of study on the level of attitude (**p = < 0.001**). The majority were males 268 (64.1%), 320 (76.6%) enrolled in clinical years with mean age 21.64 ± 1.65. The governorates of Cairo, sharqia, and Alex represented the majority with frequencies of 143 (34.2%), 106 (25.4%), and 51 (12.2%) respectively. (Table 1) However, only 175 (28.5%) have received the vaccine (Fig. 3). The majority were as follows: 83.4% were males, 65.1% were enrolled in clinical years, 88.6% were from urban residences, and 98.3% were single with statistically significant differences (p = < 0.001, < 0.001, <0.001, 0.004, respectively). The governorates of Cairo, sharqia, and Alex represented the majority with frequencies of 95 (54.3%), 27(15.4%), and 21(12%) respectively (Table 3). Table 4 presents a summary of ratings to attitude questions. Attitude towards the HPV vaccine was negatively correlated with age (*r*= −0.083, *p* = 0.04) and positively correlated with HPV knowledge (*r* = 0.136, *p* = 0.001). Regarding the vaccine role in OPC prevention, the majority of medical students strongly agreed with frequencies of 288 (47%) and 144 (23.5%) respectively. Of all participants, 303 (49.4%) agreed to recommend the vaccine to male patients, 248 (40.5%) and 242 (39.5%) agreed and strongly agreed about the necessity of the HPV vaccine for medical students. Medical school lectures were the main knowledge source about HPV and its vaccine (87.1%) (Fig. 4). The major barrier to vaccination they reported was a lack of awareness (82.4%), followed by cultural beliefs (44.5%), accessibility to vaccines (42.7%), and vaccine cost (41.3%) (Fig. 5).

**Table 3:** Characteristics of the participants according to receiving HPV vaccine.

Students characteristics	Received HPV vaccine (*n* = 175)	Did not receive HPV vaccine (*n* = 438)	*p*-value
Age ➢ Mean ± SD	21.05 ± 1.74	21.98 ± 1.508	< 0.001*
Gender			< 0.001*
➢ Male	146 (83.4%)	236 (53.9%)	
➢ Female	29 (16.6%)	202 (46.1%)	
Year of Study			< 0.001*
➢ Pre-clinical	61 (34.9%)	84 (19.2%)	
➢ Clinical	114 (65.1%)	354 (80.8%)	
Region of study			< 0.001*
➢ Cairo	95 (54.3%)	83 (18.9%)	
➢ Alex	21 (12%)	53 (12.1%)	
➢ Assiut	1 (0.6%)	13 (3%)	
➢ Aswan	-	1 (0.2%)	
➢ Beheira	-	4 (0.9%)	
➢ Benha	-	-	
➢ Beni Suef	1 (0.6%)	1 (0.2%)	
➢ Damietta	3 (1.7%)	7 (1.6%)	
➢ Suez	-	1 (0.2%)	
➢ Gharbia	11 (6.3%)	57 (13%)	
➢ Ismailia	-	3 (0.7%)	
➢ Luxur	-	1 (0.2%)	
➢ Dakahlia	8 (4.6%)	68 (15.5%)	
➢ Menofia	5 (2.9%)	15 (3.4%)	
➢ Minia	-	2 (0.5%)	
➢ Port Said	2 (1.1%)	5 (1.1%)	
➢ South Sini	1 (0.6%)	-	
➢ Sharqia	27 (15.4%)	124 (28.3%)	
Current residence			< 0.001*
➢ Urban	155 (88.6%)	312 (71.2%)	
➢ Rural	20 (11.4%)	126 (28.8%)	
Marital status			0.004*
➢ Single	172 (98.30%)	392 (89.50%)	
➢ Engaged	3 (1.70%)	43 (9.80%)	
➢ Married	0 (0.00%)	2 (0.50%)	
➢ Divorced	0 (0.00%)	1 (0.20%)

**Table 4:** Attitude towards HPV vaccine.

	Strongly agree	Agree	Neutral	Disagree	Strongly disagree
To what extent do you believe the HPV vaccine is effective in preventing oropharyngeal cancer in men?	144 (23.5%)	288 (47%)	151 (24.6%)	20 (3.3%)	10 (1.6%)
How willing are you to recommend the HPV vaccine to male patients?	181 (29.5%)	303 (49.4%)	116 (18.9%)	11 (1.8%)	2 (0.3%)
To what extent do you think HPV vaccination should be mandatory for medical students?	242 (39.5%)	248 (40.5%)	98 (16%)	19 (3.1%)	6 (1%)

## Discussion

The main cause of sexually transmitted infections (STIs), HPV-related oral pharyngeal candidacies (OPC), and their immunization program were under evaluation in this study among Egyptian medical students. Many Arab countries have divisive problems with sexually transmitted infections (STIs), which can cause social shame^21^. Since it is in other Arab nations, it can be difficult even for medical professionals to discuss HPV infections and STIs in Egypt. This could perhaps compromise their acceptance of the HPV vaccination^22^. Regular review of the medical school courses is also quite important. Given that patients with oral lesions are more likely than others to visit doctors rather than dentists, it is crucial to evaluate medical practitioners’ degree of knowledge on HPV-related oropharyngeal cancer. Consequently, it is imperative to evaluate the applicability of the training for the next generations of doctors^23^. Based on the gathered data, a considerable number of Egyptian medical students enrolled in our study with an average age of 21.72 ± 1.6, particularly single urban male students enrolled in clinical years in Cairo, Sharqia, and Gharbia governorates. In our study, the majority of participants reported that they had heard of HPV before, but only 2.1% had good knowledge. This can be explained by the existence of misconceptions or negative opinions regarding sexually transmitted diseases in Egyptian society could result in a restricted knowledge of the fact that HPV is a risk factor for OPSCC. The majority of those with limited awareness were urban single males with a mean age of 21.72 (1.6), enrolled in clinical years from the Governorates of Cairo, Sharqia, and Gharbia. Furthermore, contributing to low knowledge on this issue could be inadequate healthcare infrastructure and sociocultural limitations. Though preclinical students now have much better knowledge, the general level of knowledge was shockingly low. According to the study, limited awareness about HPV was mostly caused by the diversity of HPV kinds. Particularly about gathering patient history, which includes questions concerning sexually transmitted infections (STIs) and possible cases of sexual abuse, clinical training should take outcome into account. Notably, the number of HPV types showed a significant knowledge defect, with the main focus on choosing the most common strains only (16/18). Out of all the students involved in the study, just 7.7% knew about the link between HPV and OPC. As expected, cervical cancer came in the top answers of medical students. This can be explained by the little attention paid to HPV in local research, which has mostly concentrated on women and examined cervical cancer^24,25^.

Regarding the HPV vaccine, 92% of participants have heard about the HPV vaccine. Generally, the HPV vaccination was viewed significantly positively, especially among urban male students enrolled in our study with an average age of 21.64 ± 1.65 who were now in clinical years mostly in Cairo, Sharqia, and Alex governorates. The rather few public health education campaigns specifically addressing this issue can help to clarify it. Though the HPV vaccination is cheap in Egypt for those who would rather pay, only 28.5% have received it. The majority were single urban males, enrolled in clinical years, from the governorates of Cairo, sharqia, and Alex with a mean age of 21.05 ± 1.74. Interestingly, 70.5% of the study participants think it prevents oropharyngeal cancer. Of all participants, only 2.12% disagreed and strongly disagreed to recommend the vaccine to male patients. In our study, there was indeed a statistically significant association between knowledge of the HPV vaccine and positive attitudes towards vaccination. The Pearson’s correlation coefficient measured this relationship in our study. These results indicate a strong positive correlation: (*r* = 0.136, *p*= 0.001), which means that an increase in the level of knowledge is associated with an increase in the level of positive attitudes regarding vaccination. This finding is supported by the literature, which indicates that knowledge plays an important role in developing health-related attitudes. For example, it has been reported that higher knowledge scores are associated with more favorable attitudes toward vaccines, hence affecting the intention to vaccinate^26,27^. By explaining this relationship, we would like to stress that educational interventions based on increasing knowledge about HPV and its vaccine will be able to create more positive vaccination attitudes among medical students. For instance, the students enrolled in our study claimed that lack of awareness (82.4%), followed by cultural attitudes (44.5%), vaccination accessibility (42.7%), and vaccination cost (41.3%) are the main impediments to vaccination. **(**Fig. 5**)**Regarding cultural barriers, cultural narratives surrounding vaccine safety also play a role. Concerns about vaccine ingredients—such as porcine components in some vaccines—are particularly prevalent in Muslim populations, leading to reluctance to accept vaccinations that conflict with dietary laws^28^. Furthermore, receiving certain vaccinations, including those for sexually transmitted diseases like HPV, may be socially stigmatized in some societies. Because of these stigmas, those affected may choose not to be vaccinated out of fear of being judged or of the believed implications of their sexual activity^28^. One important factor influencing vaccine uptake is access to healthcare providers. Reduced access to medical professionals who can deliver vaccines can result in decreased immunization rates in underserved or rural locations^29^Attitudes are also greatly influenced by the caliber and accessibility of vaccine education materials. People who don’t have access to trustworthy information are more likely to believe false information and might not be completely aware of the advantages of vaccination or the dangers of diseases that can be prevented by vaccination^29,30^. The expense of vaccinations and the absence of insurance coverage are two examples of financial obstacles that may discourage people from being vaccinated. For other populations, access to vaccines might be made even more difficult by logistical issues like transportation, even when they are provided for free through public health efforts^29^.

According to present survey studies, knowledge about HPV is lacking and the relationship between HPV and oral/oropharyngeal cancer is misinterpreted^31–34^. According to a recent survey among Ukrainian medical and non-medical students, none of any student group had enough knowledge of oral or rectal cancers linked with HPV^35^. According to a recent study among Chinese female college students, HPV’s capacity to cause oral cancer was also rather unknown to them. Parsel et al.‘s recent systematic review^36^found that healthcare professionals lacked enough awareness of oropharyngeal cancer linked with HPV. That study underlined the need for extra education meant to raise knowledge of HPV-related oropharyngeal cancer. Early detection and prevention of diseases can be much influenced by this kind of comprehensive knowledge^37^.

Costa et al.‘s recent study on Brazilian medical students also noted this phenomenon. Egypt’s Human Papillomavirus and Related Cancers Fact Sheet 2023 states that the ICO/IARC Information Center on HPV and Cancer does not yet run any HPV vaccination campaigns^38^. This study suggests that female students have awareness about HPV better than male students. This can be justified by the main focus of preventive actions on cervical cancer and the importance of female immunization in preventing cervical and ovarian cancer. Apart from awareness campaigns, carefully planned training sessions and programs will help to close the knowledge gap on HPV vaccinations among Egyptian medical students, other college students, and the general population. Medical school lectures—the most often cited source of HPV knowledge—have content, approach, and teaching style we can improve. Such steps will significantly lower the prevalence of diseases connected to HPV. We strongly support more investigation on pertinent theoretical and practical teaching strategies that can fully inform medical students about HPV-related cancers and their prevention^39^.

## Limitations and recommendations

The main strength of the study was a sample size of nearly all medical students from Egyptian institutions in most governorates. Furthermore, concentrating on medical students will help to provide vital information on the knowledge and attitudes of future healthcare professionals, which is crucial for public health program planning and public education campaigns. One cheap way to get information is by using questionnaires. Answers to anonymous questionnaires can inspire individuals to be honest while reducing social desirability bias. These findings, despite the shortcomings of the research, provide us with a good estimate of the knowledge of oropharyngeal cancer connected with HPV among Egyptian medical students. Still, this questionnaire might not quite represent the knowledge of every Egyptian medical student. A cross-sectional design for the study may introduce several potential biases. First, Selection bias can occur when a sample of medical students is not representative of the population. Some universities and socioeconomic backgrounds may be overrepresented. Given that survey respondents are more likely to be those with strong opinions or knowledge of HPV, response bias may also arise. Social desirability bias is another issue; rather than expressing their true opinions or knowledge, participants may choose responses they believe to be more socially acceptable or consistent with public health messaging. Lastly, the study’s cross-sectional design restricts the ability to draw inferences on causality, as the data only provide a snapshot of attitudes and knowledge at a specific moment in time, which may alter over time or be impacted by recent events or interventions. Social desirability bias or recollection bias are among the biases that might distort responses and so compromise the data’s accuracy. For instance, several factors contribute to the fact that such findings may not be generalizable to the diverse demographics or regions in Egypt. First, medical students have received more formal education and greater exposure to healthcare topics compared to the general population, reasons for which their awareness level might be very high and thus reflect more positive attitudes toward the vaccine. This could therefore create a gap in knowledge and attitude between medical students and other groups, such as the general public, other healthcare professionals, or individuals in rural or less urbanized areas. Moreover, there might be regional differences in access to health care, cultural attitudes, and locally operated awareness campaigns that influence how different populations perceive and understand the HPV vaccine. Thus, students from major cities such as Cairo may have better access to information and healthcare services than students from more rural or underserved communities, where misinformation or lack of awareness could be greater. Extrapolating the results of this study to other populations or regions within Egypt must therefore be approached with caution, as these variables may not necessarily generalize well to the larger population. Furthermore, unlike a questionnaire, which does not always allow for in-depth research, interviews or focus groups could be more suited to capturing complicated attitudes and knowledge. Negative answers could compromise the validity of the research, causing non-response bias. People may also misinterpret questions and respond incorrectly. Cross-sectional questionnaire studies can identify relationships but cannot establish whether knowledge or attitudes lead to behaviors or outcomes.

## Conclusion

Particularly among urban male students enrolled in clinical years in the governorates of Cairo, Sharqia, and Gharbia, this study underlined the low degree of knowledge regarding HPV-related oropharyngeal cancer among Egyptian medical students. While preclinical students’ knowledge had improved dramatically, the limited awareness level -especially among clinical students- was alarming. Such findings would be related only to the population of samples in our study and cannot be generalized to all medical students in different institutions or regions. Furthermore, examining medical practitioners’ knowledge of HPV-related oropharyngeal cancer is particularly important since patients with oral lesions are more likely to consult doctors than dentists.

## Data Availability

The relevant authors of the current study have kindly provided the data sets under analysis upon appropriate demand from the corresponding author.
